# Supplementary material for: Neutralising Effects of Different Antibodies on Clostridioides difficile Toxins TcdA and TcdB in a Translational Approach
Source: Int J Mol Sci. 2023 Feb 15;24(4):3867. doi: 10.3390/ijms24043867 (PMC9962434; doi:10.3390/ijms24043867)
Supplement: Supplementary file 1 [file ijms-24-03867-s001.zip › ijms-2139469-supplementary.pdf]

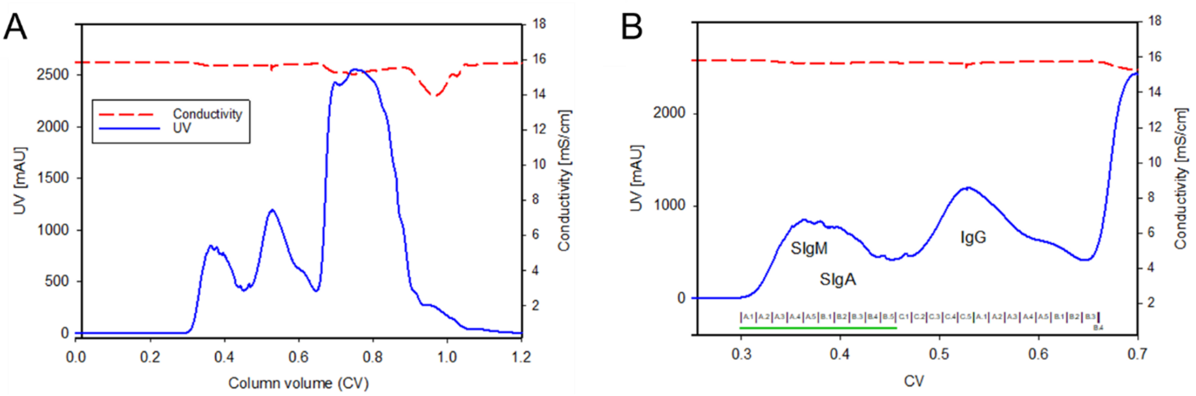

Supplementary Figure S1: Preparative size exclusion chromatography of concentrated caprine whey. (A) Complete elution profile. The first peak represents sIgM/sIgA fraction; the second peak IgG; and the third peak other whey proteins, e.g., lactoferrin, albumin, lactoglobulin and lactalbumin. (B) Zoom-in with the distribution of sIgM and sIgA based on SDS-PAGE analysis.

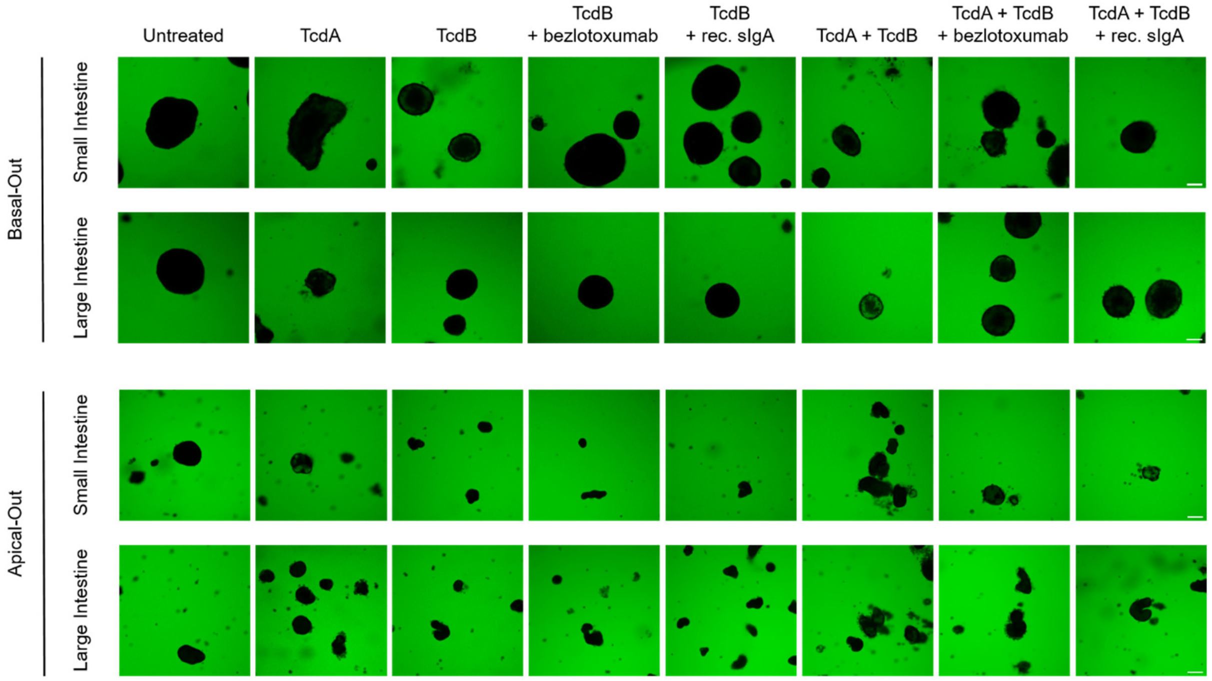

Supplementary Figure S2: Representative images from FITC-dextran barrier integrity assays using canine basal-out and apical-out organoids. Scale bars apply to all images and are 100 μm in size.
